# An MRTF-A–Sp1–PDE5 Axis Mediates Angiotensin-II-Induced Cardiomyocyte Hypertrophy

**DOI:** 10.3389/fcell.2020.00839

**Published:** 2020-09-04

**Authors:** Teng Wu, Huidi Wang, Xiaojun Xin, Xiaojun Xin, Tianyi Zhang, Yannan Hou, Mingming Fang, Xiang Lu, Yong Xu

**Affiliations:** ^1^Key Laboratory of Targeted Intervention of Cardiovascular Disease and Collaborative Innovation Center for Cardiovascular Translational Medicine, Department of Pathophysiology, Nanjing Medical University, Nanjing, China; ^2^Laboratory Center for Experimental Medicine, Department of Clinical Medicine, Jiangsu Health Vocational College, Nanjing, China; ^3^Department of Geriatrics, Sir Run Run Hospital, Nanjing Medical University, Nanjing, China; ^4^Institute of Biomedical Research, Liaocheng University, Liaocheng, China

**Keywords:** cardiac hypertrophy, cardiomyocyte, transcriptional regulation, angiotensin II, MRTF-A, Sp1, PDE5

## Abstract

Cardiac hypertrophy is a critical intermediate step in the pathogenesis of heart failure. A myriad of signaling networks converge on cardiomyocytes to elicit hypertrophic growth in response to various injurious stimuli. In the present study, we investigated the cardiomyocyte-specific role of myocardin-related transcription factor A (MRTF-A) in angiotensin-II (Ang-II)-induced cardiac hypertrophy and the underlying mechanism. We report that conditional MRTF-A deletion in cardiomyocytes attenuated Ang-II-induced cardiac hypertrophy in mice. Similarly, MRTF-A knockdown or inhibition suppressed Ang-II-induced prohypertrophic response in cultured cardiomyocytes. Of note, Ang II treatment upregulated expression of phosphodiesterase 5 (PDE5), a known mediator of cardiac hypertrophy and heart failure, in cardiomyocytes, which was blocked by MRTF-A depletion or inhibition. Mechanistically, MRTF-A activated expression of specificity protein 1 (Sp1), which in turn bound to the PDE5 promoter and upregulated PDE5 transcription to promote hypertrophy of cardiomyocytes in response to Ang II stimulation. Therefore, our data unveil a novel MRTF-A–Sp1–PDE5 axis that mediates Ang-II-induced hypertrophic response in cardiomyocytes. Targeting this newly identified MRTF-A–Sp1–PDE5 axis may yield novel interventional solutions against heart failure.

## Introduction

Cardiovascular disease (CVD) is the leading cause of deaths worldwide, posing significant health risk and socioeconomic burdens (Cardoso and Nasir, 2019). Heart failure, defined as the inability of the heart to pump blood throughout the body to sustain life activities, is one of the most devastating consequences of CVD (McMurray and Stewart, 2000). A myriad of factors, including hypertension, diabetes, and structural cardiomyopathy, contribute to heart failure (Ziaeian and Fonarow, 2016). Cardiac hypertrophy represents a common intermediate step toward heart failure regardless of etiology (Nakamura and Sadoshima, 2018). Cardiac hypertrophy is initiated as a compensatory response to offset the impairment of heart function following various injurious stimuli. Prolonged and uncontrolled hypertrophic response, however, further dampens heart function ultimately leading up to heart failure.

The pathogenesis of cardiac hypertrophy, as a prelude to heart failure, is regulated by a complex network of signaling molecules and transcription factors (Heineke and Molkentin, 2006). In response to pressure overload, a common stimulus of cardiac hypertrophy, circulating levels of angiotensin II (Ang II), epinephrine, and norepinephrine are elevated. The hormones then bind to their membrane-bound receptors, characterized by the seven-transmembrane topology and coupling to G proteins, to trigger signaling cascades (Adzika et al., 2019). On the other hand, there are counteracting signaling pathways that antagonize cardiac hypertrophy. For instance, cyclic guanosine monophosphate (cGMP)–nitric oxide (NO) can be exploited by cardiomyocytes to defy the prohypertrophic response triggered by Ang II (Booz, 2005). Cellular cGMP levels are regulated by two opposing groups of enzymes, the guanosine cyclases (GCs) and the phosphodiesterases (PDEs) (Hofmann, 2018). Accordingly, manipulations of these enzymes, either genetically or pharmacologically, contribute differentially to the pathogenesis of cardiac hypertrophy (Lukowski et al., 2014).

Myocardin-related transcription factor A (MRTF-A), also called megakaryocytic leukemia 1 (MKL1), is a transcriptional regulator with expression detected in a wide range of tissues and cells (Wang et al., 2002). MRTF-A is dispensable for the development of cardiovascular system as evidenced by the observation that mice with germline deletion of MRTF-A were born at Mendelian ratios and exhibit no overt phenotype at birth (Sun et al., 2006). Recent investigations have revealed that MRTF-A deletion or inhibition may protect the mice from CVDs including atherosclerosis (Minami et al., 2012) and cardiac ischemia–reperfusion injury (Yu et al., 2018). Previously, Kuwahara et al. (2010) have reported that systemic deletion of MRTF-A attenuated cardiac hypertrophy in mice, although it was undetermined whether the ability of MRTF-A to regulate cardiac hypertrophy was cardiomyocyte autonomous. Of note, we have since shown that endothelial-specific depletion of MRTF-A was sufficient to dampen angiotensin-II (Ang-II)-induced cardiac hypertrophy in mice (Weng et al., 2015), suggesting that at least part of the mechanism whereby MRTF-A regulates cardiac hypertrophy can be attributed to non-cardiomyocytes. Here, we report that mice with cardiomyocyte-specific MRTF-A deletion exhibit amelioration of cardiac hypertrophy. MRTF-A activates transcription of specificity protein 1 (Sp1), which in turn binds to the phosphodiesterase 5 (PDE5) promoter and upregulates PDE5 expression to promote hypertrophy of cardiomyocytes.

## Materials and Methods

### Animals

All animal procedures were reviewed and approved by the intramural Committee on Ethical Conduct of Animal Studies of Nanjing Medical University and in accordance with the National Institutes of Health (NIH) Guidelines for the Care and Use of Laboratory Animals. Mrtfa-flox mice (Yang et al., 2020) and Myh6-Cre mice (Yu et al., 2018) were crossed to generate cardiomyocyte-specific MRTF-A knockout mice. The mice were maintained under the specific pathogen-free (SPF) environment with 12 h light/dark cycles and *ad libitum* access to food and water. Male, 8-week-old MRTF-A conditional knockout (CKO) mice and wild-type (WT) littermates were induced to develop cardiac hypertrophy by angiotensin II (1 μg/kg/min, Sigma, United States) infusion for four consecutive weeks using subcutaneously implanted minipumps (Alzet, 2004) as previously described (Li et al., 2020a,b).

### Cardiac Function Assessment by Echocardiography

A non-invasive transthoracic echocardiographic examination was performed after finished angiotensin II or vesicle infusion by using a Vevo 2100 (Visualsonics, Canada), equipped with a 30-MHz transducer. The mice were anesthetized by intraperitoneal injection of pentobarbitol sodium. Two-dimensional guide M-mode tracings were recorded, and the internal diameter of the LV or left ventricular systolic dimension (LVSd), left ventricular posterior wall dimension (LVPWd), ejection fraction (EF), and fractional shortening (FS) were measured or further calculated.

### Histological Staining

Histological analyses were performed essentially as previously described (Zhao et al., 2019; Dong et al., 2020; Mao et al., 2020). Paraffin sections of heart tissue from the mice receiving different treatments were incubated with anti-MRTF-A primary antibody (1:100 dilution, Proteintech, China) and anti-actinin primary antibody (1:100 dilution, Abcam, United Kingdom) overnight at 4°C, then incubated with af594-labeled secondary antibody (1:100 dilution, Life Technologies, United States) for actinin and AF488-labeled secondary antibody (1:100 dilution, Life Technologies, United States) for MRTF-A at room temperature for 1 h. After stained with 4′,6-diamidino-2-phenylindole (DAPI) to label nuclei, the samples were observed under a confocal microscope (LSM 710, Zeiss, Germany).

For wheat germ agglutinin (WGA) staining detection, immunofluorescence (IF) staining was performed using anti-WGA (Sigma, United States) for 2 h at room temperature, Fluorescence microscopy images were obtained with a DP73 microscope (Olympus, Japan).

Paraffin sections of heart from the mice receiving different treatments were incubated with anti-MRTF-A primary antibody (1:100 dilution, Proteintech, China), anti-Sp1 primary antibody (1:100 dilution, Abcam, United Kingdom) and anti-PDE5 primary antibody (1:100 dilution, Proteintech, China), antibrain natriuretic peptide (anti-BNP) primary antibody (1:100 dilution, BOSTER, China), and anti-β-major histocompatibility complex (anti-β-MHC) primary antibody (1:100 dilution, Proteintech, China) overnight at 4°C, then incubated with CRP-labeled secondary antibody (1:100 dilution, Life Technologies, United States) at room temperature for 1 h. Positive immunostaining was visualized by using the diaminobenzidine substrate (DAB, Thermo, United States) for 1.5 min; then, hematoxylin was utilized to stain nuclei for 30 s. The samples were observed under a DP73 microscope (Olympus, Japan).

### Cell Culture, Treatment, and Transfection

The rat cardiomyocyte cell H9C2 (CRL-1446, ATCC, United States) was maintained in Dulbecco’s modified Eagle’s medium (DMEM) supplemented with 10% fetal bovine serum (FBS) at 37°C in a 5% CO_2_ incubator. Expression constructs for Sp1 (Sun et al., 2013) and PDE5A (Zhang et al., 2008) have been described previously. Small interfering RNA sequence for MRTF-A is UGGAGCUGGUGGAGAAGAA. Transient transfection was performed with Lipofectamine 2000 (Invitrogen, United States). Cells were harvested 48 h after transfection. Angiotensin II was purchased from Sigma (United States). CCG-1423 was purchased from Selleck (China). H9C2 were seeded at 1 × 10^5^ cells/p35 culture dish and starved in serum-free DMEM overnight. Angiotensin II (1 μM) was added the next day for another 12 or 24 h as previously described (Kuwahara et al., 2010). In certain experiments, CCG-1423 (10 μM) was added together with Ang II as previously described (Yu et al., 2018).

### RNA Isolation and Real-Time PCR

RNA was extracted with the RNeasy RNA isolation kit (Qiagen) as previously described (Lu et al., 2019; Shao et al., 2019; Weng et al., 2019; Yang et al., 2019a,b; Fan et al., 2020). Reverse transcriptase reactions were performed as previously described using a SuperScript First-Strand Synthesis System (Invitrogen, United States) (Zeng et al., 2018). Real-time quantitative PCR (qPCR) reactions were performed in triplicate wells on an ABI STEPONEPlus (Life Tech, United States) with the following primers: Rat *Mrtfa*, 5′-CACTCATCAAGCAAAGCCAAC CGA-3′ and 5′-AACTTCAGCTCCTGCTTCAGCTCT-3′; Rat *Pde5a*, 5′-CCCTGGCCTATTCAACAACGG-3′ and 5′-ACGTG GGTCAGGGCCTCATA-3′; Rat *Bnp*, 5′-CAGAGCTGGGGAAA GAAGAG-3′ and 5′-CCTCTGGCGGTAATAGGTGTAAAT-3′; Rat *Pde5a*, 5′-GCCCCAAATGCAGCCAT-3′ and 5′-CGCTCAG TCATGGCGGAT-3′; and mouse *Pde5a*, 5′-CGGCCTACCTGG CATTCTG-3′ and 5′-GCAAGGTCAAGTAACACCTGATT-3′. The relative quantification for a given gene was normalized by the *Gapdh* messenger RNA (mRNA) values.

### Protein Extraction and Western Blotting

Whole cell lysates were obtained by resuspending cell pellets in radioimmunoprecipitation assay (RIPA) buffer with freshly added protease inhibitor tablet (Roche, Switzerland) as previously described (Li et al., 2019a,b,c,d,e; Liu et al., 2019). Thirty micrograms of protein samples was separated by 10% sodium dodecyl sulfate–polyacrylamide gel electrophoresis (SDS-PAGE) electrophoresis and transferred to nitrocellulose membranes (Millipore, United States). Western analyses were performed with anti-α-tubulin (Proteintech, Wuhan, China 11224-1, 1:2,000), anti-MRTF-A (Proteintech, China 21166-1, 1:1,000), anti-PDE5 (Proteintech, China 22624-1, 1:1,000), anti-BNP primary antibody (BOSTER, China, 1:1,000 dilution), anti-β-MHC primary antibody (Proteintech, China, 1:1,000 dilution), and anti-Sp1 (Abcam, United Kingdom ab13370, 1:1,000). Image J software was used for densitometrical quantification, and densities of target proteins were normalized to those of α-tubulin. Data are expressed as relative protein levels compared to the control group, which is arbitrarily set as 1.

### Immunofluorescence Staining

Immunofluorescence staining was performed as previously described (Fan et al., 2019; Kong et al., 2019a,b). Briefly, H9C2 cells were plated at a density of 2 × 10^4^ cells per dish. After treatment with Ang II, the cells were washed with phosphate-buffered saline (PBS) three times, fixed by 4% paraformaldehyde for 5 min, and stained with an α-actinin antibody (Sigma, United States A7811, 1:200) overnight at 4°C. The next day, the cells incubated with AF488-labeled secondary antibody (Jackson ImmunoResearch) for 1 h. The nuclei were counterstained with DAPI (Sigma, United States). Immunofluorescence was visualized on a confocal microscope (LSM 710, Zeiss).

### Electrophoresis Mobility Shift Assay

Nuclear protein extraction was performed essentially as previously described. Briefly, cells were resuspended in the cytoplasmic lysis buffer [140 mM NaCl, 10 mM HEPES, pH 7.4, 1 mM ethylenediaminetetraacetic acid (EDTA), 1.5 mM MgCl_2_, 0.5 mM dithiothreitol (DTT)] and incubated for 15 min. After centrifugation, the supernatant was collected and labeled as the cytoplasmic fraction. The pellet was resuspended in the nuclear lysis buffer (450 mM NaCl, 1 mM EDTA, 20 mM HEPES, pH 7.4, 0.5 mM DTT) and incubated for 15 min. After centrifugation, the supernatant was collected and labeled as the nuclear fraction. The nuclear proteins (5 μg) were incubated with 1 × binding buffer (LightShift Chemiluminescent EMSA Kit, Pierce) in the presence of 50 ng/μl poly(dI/dC), 0.05% non-idet P-40, 5 mM MgCl_2_, and 2.5% glycerol for 10 min and then incubated at room temperature for additional 20 min with 1 pmol of biotin-labeled Sp1 oligonucleotide (Sangon Biotech Co., Ltd.). For supershift assay, 1 μg anti-Sp1 antibody was added to the reaction mixture. The DNA–protein complex was subjected to a 6% non-denaturing SDS-PAGE at 100 V for 60 min, transferred to polarity nylon hybridization membrane (Beyotime, China), and UV cross-linked for 10 min. After the addition of peroxidase-conjugated streptavidin antibodies (Beyotime), visualization was achieved on a ChemiDoc Image Station (Bio-Rad). The sequence for wild-type Sp1 probe is 5′-ATTTGTTCG**GGGCGG**GGCGAGC-3′ and that for mutant Sp1 probe is 5′-ATTTGT TCG**GTTCGG**GGCGAGC-3′.

### Transfection and Luciferase Report Assay

293T cells were transfected using Lipofectamine 2000 (Invitrogen, United States) in serum-free DMEM media with Sp-1-Luc (−1,500/ + 50) and different concentrations (0.2, 0.5, and 1 μg) of MRTF-A overexpression plasmid or transfected with PDE5-Luc (−2,000/+50) and different concentrations (0.2, 0.5, and 1 μg) of Sp-1 overexpression plasmid. Six hours after transfection, the cells were cultured in DMEM containing 10% FBS for 24 h, the cells were lyzed, and luciferase activity was determined using the luciferase assay system according to the manufacture’s instruction (Promega Corp., United States). For controlling for differences in transfection efficiency, a green fluorescent protein (GFP) plasmid was included in each transfection and used for normalization.

### Neonatal Rat Ventricular Myocytes Separates

Neonatal rat ventricular myocytes were prepared from 1- to 2-day-old neonatal Sprague Dawley rats, the cells were incubated in DMEM medium (Gibco, United States) supplemented with 10% fetal bovine serum. To prevent proliferation of cardiac fibroblasts (CFs), bromodeoxyuridine (0.1 mM) was added into cultured neonatal rat ventricular myocytes. Angiotensin II and MRTF-A small interfering RNA (siRNA) or CCG treatment protocol focus on the statement above.

### Statistical Analysis

Data are presented as mean ± SD. For experiments concerning multiple groups, one-way ANOVA with *post hoc* Scheffe analyses was performed to evaluate the differences using an SPSS package (IBM analytics). The differences between the two (control and experimental) groups were determined by two-sided, unpaired Student’s *t* test.

## Results

### Cardiomyocyte Conditional Deletion of MRTF-A Attenuates Ang-II-Induced Cardiac Hypertrophy *in vivo*

**FIGURE 1:**
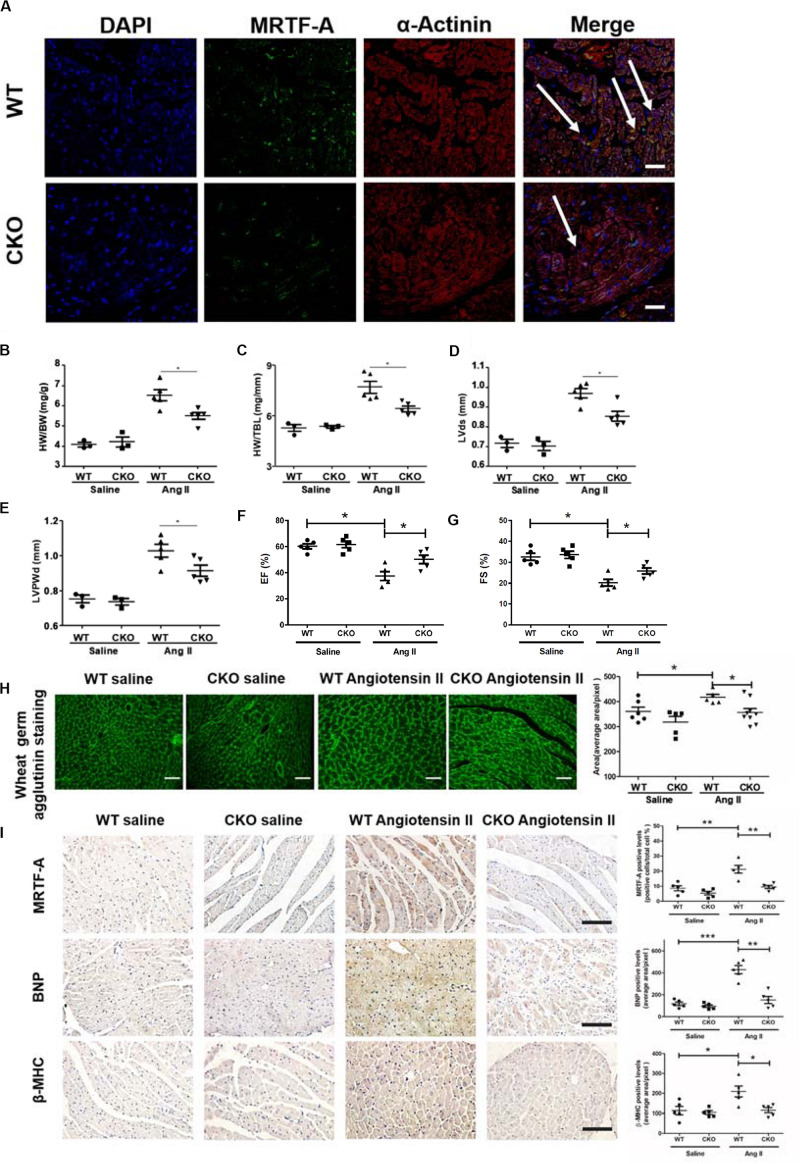
Cardiomyocyte deletion of myocardin-related transcription factor A (MRTF-A) attenuates Ang-II-induced cardiac hypertrophy in mice. MRTF-A conditional knockout (CKO) mice and wild-type (WT) mice were induced to develop cardiac hypertrophy by Ang II infusion as described in section “Materials and Methods.” **(A)** Immunofluorescence staining was performed with anti-MRTF-A and anti-actinin. **(B)** Heart weight vs. body weight ratio. **(C)** Heart weight vs. tibia bone length ratio. **(D)** Echocardiographic measurements of left ventricular systolic dimension (LVSd). **(E)** Echocardiographic measurements of left ventricular posterior wall dimension (LVPWd). **(F)** Echocardiographic measurements of ejection fraction (EF). **(G)** Echocardiographic measurements of fractional shortening (FS). **(H)** Cardiac sections were stained with wheat germ agglutinin (WGA). Cross-sectional areas were quantified by Image Pro. **(I)** Immunohistochemistry (IHC) staining of cardiac sections with anti-MRTF-A, antibrain natriuretic peptide (anti-BNP), and anti-β-major histocompatibility complex (anti-β-MHC) antibodies, Cross-sectional areas were quantified by Image Pro. *N* = 3–5 mice for the saline groups and *N* = 5 mice for the Ang II groups. Error bars represent SD. *p < 0.05; **p < 0.01; ***p < 0.001. Scale bar: 50 μm.

Previously, it has been demonstrated that systemic deletion of MRTF-A attenuates pathological cardiac hypertrophy in mice (Kuwahara et al., 2010). In order to assign a cell-specific role for MRTF-A in the pathogenesis of cardiac hypertrophy, MRTF-A was specifically deleted in cardiomyocytes by removing the floxed *Mrtfa* allele (Liu et al., 2018) with a *Myh6*-Cre driver (Yu et al., 2018). Immunofluorescence staining by MRTF-A and α-actinin identified lower MRTF-A expression in MRTF-A CKO cardiomyocytes (Figure 1A). The CKO mice appeared indistinguishable from the WT littermates and did not exhibit any overt phenotype under physiological conditions (data not shown). Cardiac hypertrophy was induced in CKO mice and the control mice (WT) by chronic Ang II infusion for 4 weeks. Ang II infusion provoked robust hypertrophic response in the murine hearts as evidenced by elevated heart weight/body weight ratios (Figure 1B) and heart weight/tibia bone length ratios (Figure 1C); the CKO hearts displayed a much weaker hypertrophic phenotype than the WT hearts. Consistent with these results, echocardiographic measurements showed that augmentation of LVSd (Figure 1D) and LVPWd (Figure 1E) following Ang II infusion was less prominent in the CKO mice than in the WT mice. On the contrary, decreases in EF (Figure 1F) and FS (Figure 1G) in WT mice were partially ameliorated in the CKO mice. Additional evidence that cardiomyocyte-specific MRTF-A deletion attenuated Ang-II-induced cardiac hypertrophy was provided by WGA staining of the heart sections: cross-sectional areas were smaller in the Ang-II-infused CKO hearts than in the Ang-II-infused WT hearts (Figure 1H). Further research via immunohistochemistry (IHC) staining demonstrated lower expression of MRTF-A, BNP, and β-MHC in the Ang-II-infused CKO hearts than in the Ang-II-infused WT hearts (Figure 1I). Taken together, these data suggest that cardiomyocyte conditional deletion of MRTF-A attenuates Ang-II-induced cardiac hypertrophy *in vivo*.

### MRTF-A Depletion or Inhibition Attenuates Ang-II-Induced Cardiomyocyte Hypertrophy *in vitro*

**FIGURE 2:**
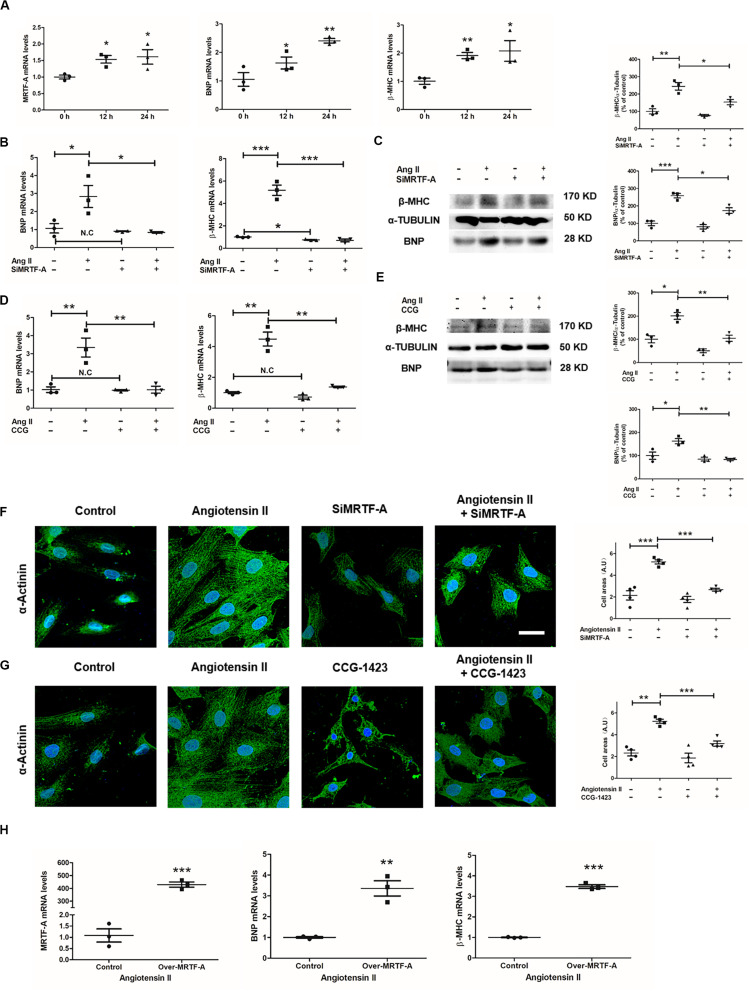
Myocardin-related transcription factor A (MRTF-A) depletion or inhibition attenuates Ang-II-induced cardiomyocyte hypertrophy *in vitro*. **(A)** H9C2 cells were treated with or without Ang II (1 μM) and harvested at indicated time points. Gene expression was examined by quantitative PCR (qPCR). **(B,C)** H9C2 cells were transfected with small interfering RNA (siRNA) targeting MRTF-A or scrambled siRNA (SCR) followed by treatment with Ang II (1 μM) for 24 h. Gene expression was examined by qPCR and Western blot. **(D,E)** H9C2 cells were treated with Ang II (1 μM) and CCG-1423 (10 μM) for 24 h. Gene expression was examined by qPCR and Western blot. **(F)** H9C2 cells were transfected with siRNA targeting MRTF-A or scrambled siRNA (SCR) followed by treatment with Ang II (1 μM) for 24 h. Immunofluorescence staining was performed with anti-α-actinin. Cross-sectional areas were quantified by Image Pro. **(G)** H9C2 cells were treated with Ang II (1 μM) and CCG-1423 (10 μM) for 24 h. Immunofluorescence staining was performed with anti-α-actinin. Cross-sectional areas were quantified by Image Pro. **(H)** H9C2 cells were treated with Ang II (1 μM) and transfected with MRTF-A expression plasmid for 24 h. Gene expression was examined by qPCR. Data represent averages of three independent experiments and error bars represent SEM. *p < 0.05; **p < 0.01; ***p < 0.001. Scale bar: 25 μm.

Next, we evaluated the effect of MRTF-A depletion or inhibition on Ang-II-induced hypertrophy in cultured cardiomyocytes. qPCR revealed that Ang II treatment induced a small but significant upregulation of MRTF-A mRNA levels, paralleling an increase in BNP and β-MHC mRNA levels, in H9C2 cells (Figure 2A). Knockdown of MRTF-A expression with siRNA suppressed the induction of BNP and β-MHC expression by Ang II (Figure 2B). Western blot also demonstrated lower BNP and β-MHC expression as a result of MRTF-A deletion (Figure 2C). A small-molecule compound CCG-1423 was also utilized to inhibit MRTF-A function in H9C2 cells (Chong et al., 2012). Similarly, inhibition of MRTF-A by CCG-1423 blocked the induction of BNP and β-MHC by Ang II at both mRNA (Figure 2D) and protein levels (Figure 2E). Accordingly, immunofluorescence staining of α-actinin, as a measurement of cell surface area, showed that both siRNA-mediated depletion of MRTF-A (Figure 2F) or CCG-mediated inhibition of MRTF-A (Figure 2G) ameliorated Ang-II-induced hypertrophy of H9C2 cells. H9C2 cells were transfected with an MRTF-A plasmid. As shown in Figure 2H, under the Ang II treatment condition, MRTF-A overexpression in H9C2 cells significantly upregulated BNP and β-MHC expression. Combined, these data suggest that MRTF-A deficiency attenuates Ang-induced cardiomyocyte hypertrophy *in vitro*.

### MRTF-A Mediates Ang-II-Induced PDE5A Expression in Cardiomyocytes

**FIGURE 3:**
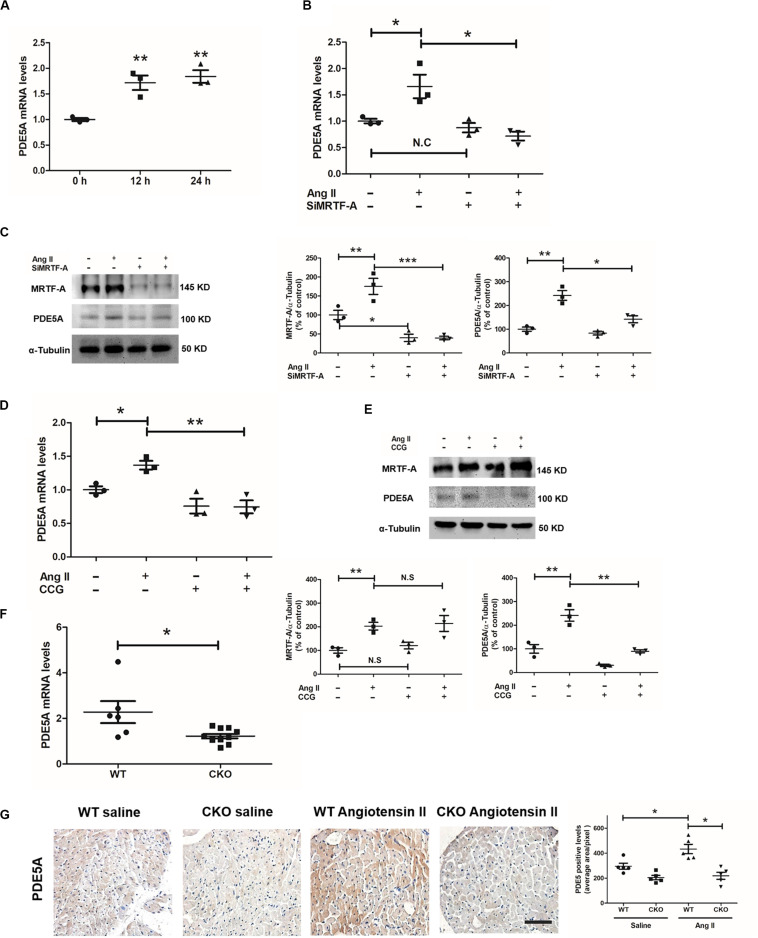
Myocardin-related transcription factor A (MRTF-A) mediates Ang-II-induced phosphodiesterase 5A (PDE5A) expression in cardiomyocytes. **(A)** H9C2 cells were treated with or without Ang II (1 μM) and harvested at indicated time points. Gene expression was examined by quantitative PCR (qPCR). **(B,C)** H9C2 cells were transfected with small interfering RNA (siRNA) targeting MRTF-A or scrambled siRNA (SCR) followed by treatment with Ang II (1 μM) for 24 h. Gene expression was examined by qPCR and Western blot. **(D,E)** H9C2 cells were treated with Ang II (1 μM) and CCG-1423 (10 μM) for 24 h. Gene expression was examined by qPCR and Western blot. **(F)** Wild-type (WT) and conditional knockout (CKO) mice were subjected to chronic Ang II infusion for 4 weeks. Cardiac PDE5A expression was examined by qPCR. **(G)** Immunohistochemistry (IHC) staining for cardiac sections with anti-PDE5A antibodies, Data represent averages of three independent experiments, and error bars represent SEM. *p < 0.05; **p < 0.01; ***p < 0.001. Scale bar: 50 μm.

Phosphodiesterase 5A has been reported to mediate the prohypertrophic response induced by Ang II in cardiomyocytes (Nagendran et al., 2007; Pokreisz et al., 2009). Indeed, Ang II treatment elicited a robust upregulation of PDE5A in H9C2 cells (Figure 3A). We asked whether MRTF-A might contribute to Ang-II-induced PDE5A expression in cardiomyocytes. In the first set of experiments, endogenous MRTF-A was depleted by siRNA. MRTF-A knockdown significantly dampened the induction of PDE5A expression, as measured by qPCR (Figure 3B) and Western blotting (Figure 3C), by Ang II treatment. In the second set of experiments, the cells were treated with CCG-1423, a known small-molecule MRTF-A inhibitor (Evelyn et al., 2007). Inhibition of MRTF-A activity by CCG treatment comparably suppressed the induction of PDE5A mRNA (Figure 3D) and protein (Figure 3E) expression by Ang II. In animal models, cardiomyocytes-specific MRTF-A knockout inhibited Ang-II-induced PDE5A expression (Figures 3F,G).

### PDE5A Is Essential for MRTF-A to Mediate the Prohypertrophic Response in Cardiomyocytes

**FIGURE 4:**
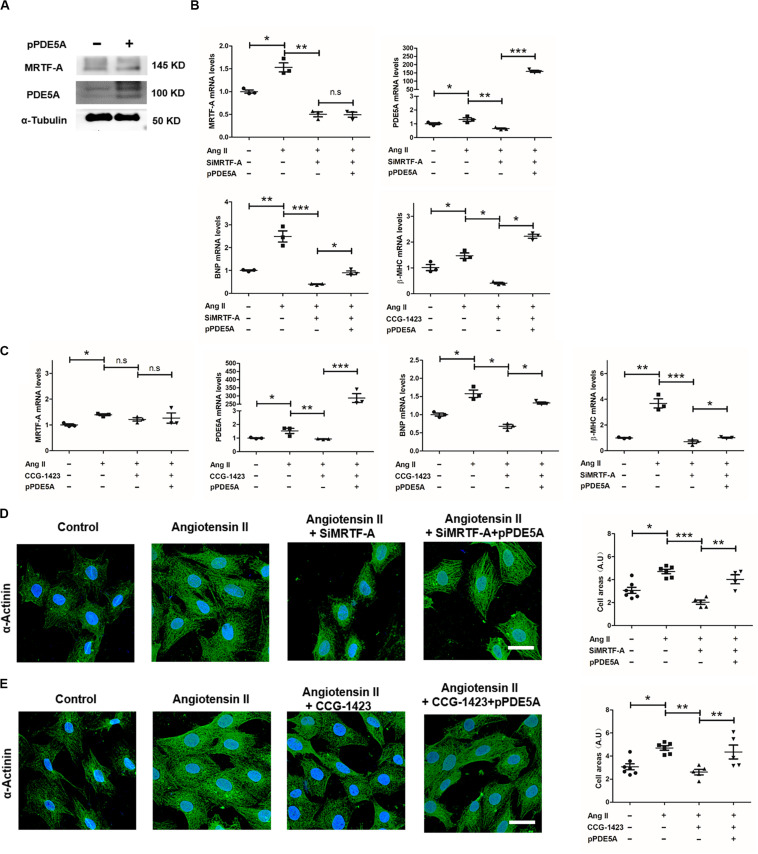
Phosphodiesterase 5 (PDE5) is essential for myocardin-related transcription factor A (MRTF-A) to mediate Ang-II-induced prohypertrophic response in cardiomyocytes. **(A)** H9C2 cells were transfected with a PDE5A vector or an empty vector. Protein expression was examined by Western blot. **(B)** H9C2 cells were transfected with small interfering RNA (siRNA) targeting MRTF-A or scrambled siRNA (SCR) in the presence or absence of ectopic PDE5A followed by treatment with Ang II (1 μM) for 24 h. Gene expression was examined by quantitative PCR (qPCR). **(C)** H9C2 cells were treated with Ang II (1 μM) and CCG-1423 (10 μM) in the presence or absence of ectopic PDE5A for 24 h. Gene expression was examined by qPCR. **(D)** H9C2 cells were transfected with siRNA targeting MRTF-A or scrambled siRNA (SCR) in the presence or absence of ectopic PDE5A followed by treatment with Ang II (1 μM) for 24 h. Immunofluorescence staining was performed with anti-α-actinin. Cross-sectional areas were quantified by Image Pro. **(E)** H9C2 cells were treated with Ang II (1 μM) and CCG-1423 (10 μM) in the presence or absence of ectopic PDE5A for 24 h. Immunofluorescence staining was performed with anti-α-actinin. Cross-sectional areas were quantified by Image Pro. Data represent averages of three independent experiments, and error bars represent SEM. *p < 0.05; **p < 0.01; ***p < 0.001. Scale bar: 25 μm.

We next asked whether PDE5A might be a downstream effector of MRTF-A that contributes to Ang-II-induced hypertrophic response in cardiomyocytes. To this end, an ectopic PDE5A vector was transfected into H9C2 cells resulting in robust upregulation of PDE5 protein levels (Figure 4A); of note, MRTF-A expression was not significantly altered by PDE5 overexpression. As shown in Figure 4B, although MRTF-A depletion by siRNA attenuated Ang-II-induced BNP and β-MHC expression, PDE5 overexpression largely restored BNP and β-MHC expression. Similarly, PDE5 overexpression also defied the effect of MRTF-A inhibition by CCG treatment and allowed BNP induction by Ang II (Figure 4C). In addition, measurements of cardiomyocyte cross-sectional areas by α-actinin immunofluorescence staining confirmed that, whereas MRTF-A knockdown (Figure 4D) or inhibition (Figure 4E) attenuated Ang-II-induced hypertrophy, reintroduction of exogenous PDE5A effectively circumvented the deficiency of MRTF-A and restored the prohypertrophic response in H9C2 cells. Together, these data suggest that PDE5A acts downstream of MRTF-A to promote Ang-II-induced cardiomyocyte hypertrophy.

### MRTF-A Activates Sp1 Expression to Stimulate PDE5A Transcription

**FIGURE 5:**
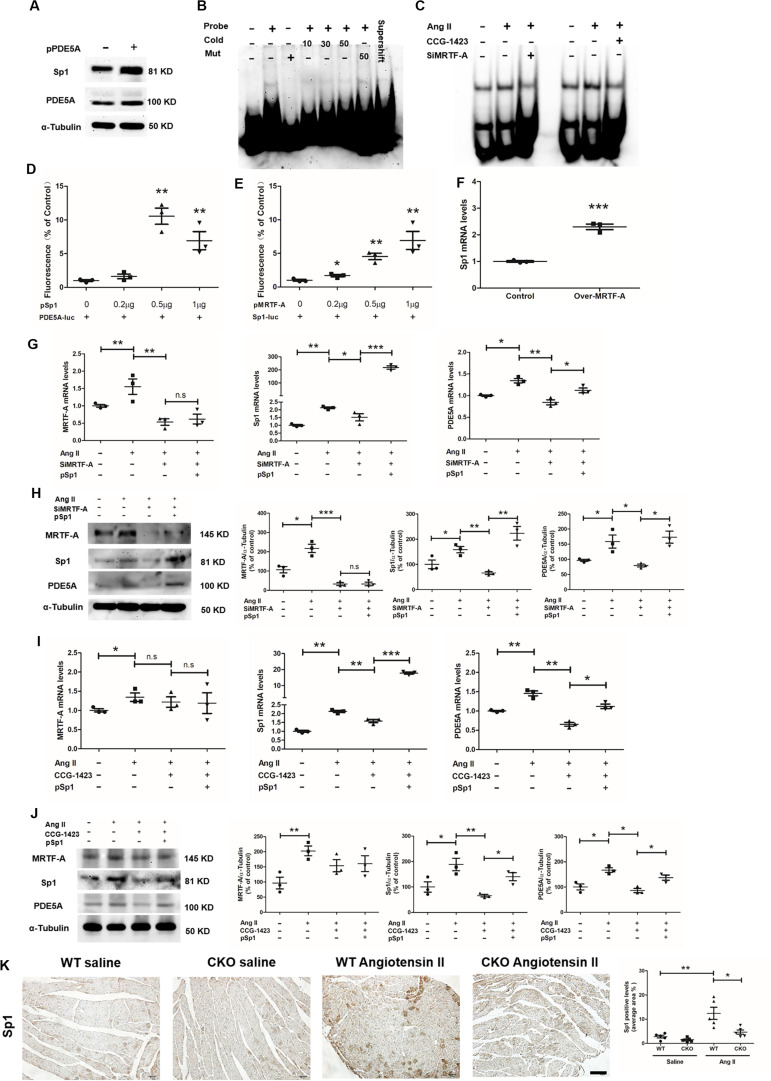
Sp1 directly activates phosphodiesterase 5A (PDE5A) transcription in cardiomyocytes. **(A)** H9C2 cells were transfected with an Sp1 vector or an empty vector. Protein expression was examined by Western blot. **(B)** Nuclear lysates were extracted from H9C2 cells, and electrophoresis mobility shift assay (EMSA) was performed as described in section “Materials and Methods.” **(C)** H9C2 cells were transfected with small interfering RNA (siRNA) targeting myocardin-related transcription factor A (MRTF-A) or scrambled siRNA (SCR) followed by treatment with Ang II (1 μM) for 24 h. Alternatively, H9C2 cells were treated with Ang II (1 μM) and CCG-1423 (10 μM) for 24 h. Nuclear lysates were extracted, and EMSA was performed as described in section “Materials and Methods.” **(D)** 293T cells were cotransfected with plasmid containing PDE5-promoter-luc and/or different concentrations of Sp1 overexpression plasmid. Luciferase report assay was performed as described in section “Materials and Methods.” **(E)** 293T cells were cotransfected with plasmid containing Sp1-promoter-luc and/or different concentrations of MRTF-A overexpression plasmid. Luciferase report assay was performed as described in section “Materials and Methods.” **(F)** H9C2 cells cotransfected with plasmid overexpression MRTF-A, Sp1 expression was examined by quantitative PCR (qPCR). **(G,H)** H9C2 cells were transfected with siRNA targeting MRTF-A or scrambled siRNA (SCR) in the presence or absence of ectopic Sp1 followed by treatment with Ang II (1 μM) for 24 h. Gene expression was examined by qPCR and Western blot. **(I,J)** H9C2 cells were treated with Ang II (1 μM) and CCG-1423 (10 μM) in the presence or absence of ectopic Sp1 for 24 h. Gene expression was examined by qPCR and Western blot. **(K)** Immunohistochemistry (IHC) staining of cardiac sections with anti-Sp1 antibodies. Data represent averages of three independent experiments, and error bars represent SEM. *p < 0.05; **p < 0.01; ***p < 0.001. Scale bar: 50 μm.

A scanning of the proximal PDE5A promoter revealed a GC-rich region resembling the conserved motif for the transcription factor Sp1. When an ectopic Sp1 vector was transfected into H9C2, it was noted that PDE5A protein levels were significantly upregulated, suggesting that Sp1 may directly activate PDE5A transcription (Figure 5A). Electrophoresis mobility shift assay (EMSA) showed that a specific band appeared when H9C2 nuclear proteins were incubated with the wild-type PDE5A promoter probe containing intact Sp1 motif but not the mutated PDE5A promoter probe (Figure 5B); this band disappeared when an anti-Sp1 was coincubated with the DNA–protein complex. Further, Ang II treatment enhanced the interaction of Sp1 with PDE5A promoter, which was blocked by either MRTF-A knockdown or MRTF-A inhibition (Figure 5C). Luciferase report assay, as shown in Figure 5D, proved that the PDE5A promoter was activated by Sp1 overexpression in 293T cells. As shown in Figure 5E, the Sp1 promoter activity was also induced by an MRTF-A overexpression plasmid in a dose-dependent manner. In addition, MRTF-A overexpression also upregulated endogenous Sp1 levels in H9C2 cells (Figure 5F). Based on these observations, we hypothesized that MRTF-A might activate the expression of Sp1, which in turn stimulated PDE5A transcription. As shown in Figures 5G,H, although MRTF-A knockdown severely compromised the ability of Ang II to induce PDE5A expression, overexpression of ectopic Sp1 compensated for the loss of MRTF-A and restored PDE5A induction by Ang II. Similarly, Sp1 overexpression rescued the repression of PDE5A upregulation by CCG treatment (Figures 5I,J). In animal models, cardiomyocytes-specific MRTF-A knockout inhibited Ang-II-induced Sp1 expression (Figure 5K).

**FIGURE 6:**
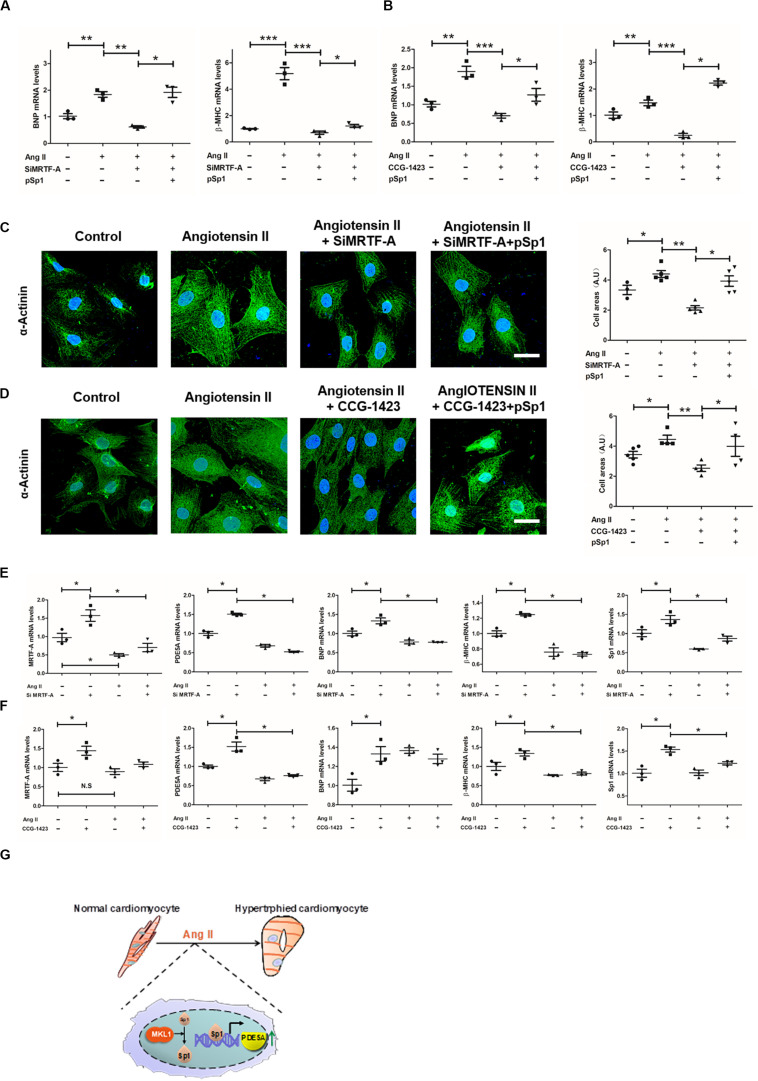
Sp1-mediated phosphodiesterase 5 (PDE5) transcription is essential for myocardin-related transcription factor A (MRTF-A) to mediate Ang-II-induced prohypertrophic response in cardiomyocytes. **(A)** H9C2 cells were transfected with siRNA targeting MRTF-A or scrambled siRNA (SCR) in the presence or absence of ectopic Sp1 followed by treatment with Ang II (1 μM) for 24 h. Gene expression was examined by quantitative PCR (qPCR). **(B)** H9C2 cells were treated with Ang II (1 μM) and CCG-1423 (10 μM) in the presence or absence of ectopic Sp1 for 24 h. Gene expression was examined by qPCR. **(C)** H9C2 cells were transfected with siRNA targeting MRTF-A or scrambled siRNA (SCR) in the presence or absence of ectopic Sp1 followed by treatment with Ang II (1 μM) for 24 h. Immunofluorescence staining was performed with anti-α-actinin. Cross-sectional areas were quantified by Image Pro. **(D)** H9C2 cells were treated with Ang II (1 μM) and CCG-1423 (10 μM) in the presence or absence of ectopic Sp1 for 24 h. Immunofluorescence staining was performed with anti-α-actinin. **(E)** Primary neonatal rat ventricular myocytes were transfected with siRNA targeting MRTF-A or scrambled siRNA (SCR) followed by treatment with Ang II (1 μM) for 24 h. Gene expression was examined by qPCR. **(F)** Primary neonatal rat ventricular myocytes were treated with Ang II (1 μM) and CCG-1423 (10 μM) for 24 h. Gene expression was examined by qPCR. **(G)** A schematic model. Data represent averages of three independent experiments, and error bars represent SEM. *p < 0.05; **p < 0.01; ***p < 0.001. Scale bar: 25 μm.

We evaluated the functional relevance of this MRTF-A–Sp1–PDE5A in Ang-II-induced cardiomyocyte hypertrophy. As shown in Figures 6A,B, Ang II strongly upregulated BNP and β-MHC expression in H9C2 cells, which was attenuated by MRTF-A depletion or MRTF-A inhibition. Forced expression of Sp1 restored the Ang-II-induced prohypetrophic response by bringing up BNP expression. Similar observations were made when α-actinin immunofluorescence staining was performed to measure cardiomyocyte cross-sectional areas (Figures 6C,D).

Finally, we also measured the function of Ang-II-induced cardiomyocyte hypertrophy in primary neonatal rat ventricular myocytes. As shown in Figures 6E,F, MRTF-A knockdown or inhibition by CCG-1423 treatment obviously inhibited upregulation of MRTF-A, Sp1, PDE5A, β-MHC, and BNP induced by Ang II treatment.

## Discussion

Pathological cardiac hypertrophy is a key pathophysiological process in the development of heart failure. A host of mechanical and neurohumoral factors can stimulate cardiac hypertrophy. MRTF-A deficiency, both systemic (Kuwahara et al., 2010) and endothelial specific (Weng et al., 2015), has been shown to attenuate cardiac hypertrophy. Our data demonstrate for the first time that cardiomyocyte-restricted deletion of MRTF-A is sufficient to alleviate Ang-II-induced cardiac hypertrophy in mice. In addition, we present evidence to show that an MRTF-A–Sp1–PDE5A axis mediates the prohypertrophic response in cardiomyocytes (Figure 6G). Although the prohypertrophic response primarily takes place in cardiomyocytes, it is not necessarily an exclusively cardiomyocyte-autonomous process. Instead, there is abundant evidence to suggest that non-cardiomyocytes, including fibroblasts, endothelial cells, epicardial cells, and blood-borne myeloid cells, play key roles in cardiac hypertrophy (Kamo et al., 2015). MRTF-A expression is widely distributed throughout the cardiovascular system (Wang et al., 2002), raising the intriguing question as to whether MRTF-A is able to direct distinct, cell-type-specific role transcriptional programs to regulate cardiac hypertrophy. Our previously published data suggest that MRTF-A activates the transcription of endothelin (ET-1), which consequently acts on the cardiomyocytes to stimulate a prohypertrophic response (Weng et al., 2015). Here, we show that MRTF-A can directly promote Ang-II-induced hypertrophic response in cardiomyocytes likely through PDE5A upregulation. However, these data do not foreclose the possibilities that fibroblast-specific MRTF-A or myeloid-specific MRTF-A may play equally important roles in cardiac hypertrophy. It has been well documented that MRTF-A is a pivotal regulator of fibroblast phenotype (Small, 2012) and myeloid phenotype (Yu et al., 2014, 2017; An et al., 2017, 2019). Future studies exploiting more lineage-specific animal models would help clarify the origins of MRTF-A-dependent prohypertrophic cues during cardiac hypertrophy.

We show that MRTF-A regulates PDE5A transcription indirectly by activating Sp1 expression. Sp1 can potentially promote cardiac hypertrophy by activating the transcription of a panel of hypertrophic genes, including troponin T (Azakie et al., 2006), ANF (Hu et al., 2011), HCN2/4 (Lin et al., 2009), and SERCA2 (Takizawa et al., 2003). In addition, Sp1, in cooperation with nuclear receptors, appears to be essential for the metabolic reprogramming during cardiac hypertrophy by orchestrating the transcription of fatty acid oxidation-related genes (Sack et al., 1997). Consistent with previous reports that Sp1 levels are upregulated in the hypertrophied hearts (Azakie et al., 2006; Hu et al., 2011), we show here that Ang II treatment stimulates Sp1 expression in cultured cardiomyocytes, a process that requires the presence of MRTF-A. It remains unknown whether MRTF-A could bind to the Sp1 promoter and activate its transcription. The proximal Sp1 promoter contains several conserved binding sites for NF-Y and E2F1 (Nicolas et al., 2003). Since both NF-Y (Foster et al., 2017) and E2F1 (Li et al., 2018) have been shown to interact with MRTF-A and recruit MRTF-A to target gene promoters, the possibility that MRTF-A may, via NF-Y and/or E2F1, directly regulate Sp1 transcription certainly deserves further attention.

Based on the observations as summarized here, a key question to ask is whether targeting this MRTF-A–Sp1–PDE5A axis would engender benefits in treating cardiac hypertrophy and heart failure. PDE5A inhibitors have been used in the clinics with proven effectiveness in improving ventricular function and reducing morality in patients with heart failure (Hutchings et al., 2018). Recently, Xiong et al. (2019) have reported that administration of CCG-1423-8u, a modified and supposedly more potent derivative of CCG-1423 as used in the present study, attenuates cardiac hypertrophy and rescues heart function in a mouse model of dilated cardiomyopathy. Similarly, Zhou et al. (2017) have shown that treatment with CCG-100602, yet another derivative of CCG-1423, ameliorates the vessel wall stiffness in a rat model of hypertension, generally considered as a cause for cardiac hypertrophy and heart failure. On the other hand, few reports have revealed the usefulness of specific Sp1 inhibitor(s) in the treatment of cardiac hypertrophy partly because Sp1 is a universally present molecule and partly because sequence-specific transcription factors are notorious to drug. Zhang et al. have recently reported that the Sp1 inhibitor mithramycin protects the mice from vascular calcification (Zhang et al., 2018), suggesting that targeting Sp1 may be associated with beneficial effects at least under specific circumstances. Our data certainly reinforce the call for screening small-molecule compounds targeting the MRTF-A–Sp1–PDE5A axis to treat cardiac hypertrophy and heart failure.

## Data Availability Statement

The raw data supporting the conclusions of this article will be made available by the authors, without undue reservation, to any qualified researcher.

## Ethics Statement

The animal study was reviewed and approved by Committee on Ethical Conduct of Animal Studies of Nanjing Medical University.

## Author Contributions

YX and TW conceived the project, designed the experiments, and wrote the manuscript. TW, HW, XX, TZ, JY, and YH performed experiments and collected data. MF and XL secured funding and provided supervision. All authors contributed to the article and approved the submitted version.

## Conflict of Interest

The authors declare that the research was conducted in the absence of any commercial or financial relationships that could be construed as a potential conflict of interest.

## References

[B1] Adzika G. K. Machuki J. O. Shang W. Hou H. Ma T. Wu L. (2019). Pathological cardiac hypertrophy: the synergy of adenylyl cyclases inhibition in cardiac and immune cells during chronic catecholamine stress. *J. Mol. Med.* 97 897–907. 10.1007/s00109-019-01790-1790 31062036

[B2] An J. Nagaishi T. Watabe T. Naruse T. K. Watanabe M. Kimura A. (2017). MKL1 expressed in macrophages contributes to the development of murine colitis. *Sci. Rep.* 7:13650. 10.1038/s41598-017-13629-13620 PMC5651926 29057966

[B3] An J. Naruse T. K. Hinohara K. Soejima Y. Sawabe M. Nakagawa Y. (2019). MRTF-A regulates proliferation and survival properties of pro-atherogenic macrophages. *J. Mol. Cell Cardiol.* 133 26–35. 10.1016/j.yjmcc.2019.05.015 31128166

[B4] Azakie A. Fineman J. R. He Y. (2006). Sp3 inhibits Sp1-mediated activation of the cardiac troponin T promoter and is downregulated during pathological cardiac hypertrophy in vivo. *Am. J. Physiol. Heart Circ. Physiol.* 291 H600–H611. 10.1152/ajpheart.01305.2005 16617124

[B5] Booz G. W. (2005). Putting the brakes on cardiac hypertrophy: exploiting the NO-cGMP counter-regulatory system. *Hypertension* 45 341–346. 10.1161/01.HYP.0000156878.17006.02 15710777

[B6] Cardoso R. Nasir K. (2019). Primary prevention of cardiovascular disease: 2019 and beyond. *Nat. Rev. Cardiol.* 16 387–388. 10.1038/s41569-019-0213-212 31110263

[B7] Chong N. W. Koekemoer A. L. Ounzain S. Samani N. J. Shin J. T. Shaw S. Y. (2012). STARS is essential to maintain cardiac development and function in vivo via a SRF pathway. *PLoS One* 7:e40966. 10.1371/journal.pone.0040966 22815879 PMC3399798

[B8] Dong W. Kong M. Zhu Y. Shao Y. Wu D. Lu J. (2020). Activation of TWIST transcription by chromatin remodeling protein brg1 contributes to liver fibrosis in mice. *Front. Cell Dev. Biol.* 8:340. 10.3389/fcell.2020.00340 32478075 PMC7237740

[B9] Evelyn C. R. Wade S. M. Wang Q. Wu M. Iniguez-Lluhi J. A. Merajver S. D. (2007). CCG-1423: a small-molecule inhibitor of RhoA transcriptional signaling. *Mol. Cancer Ther.* 6 2249–2260. 10.1158/1535-7163.MCT-06-0782 17699722

[B10] Fan Z. Kong M. Li M. Hong W. Fan X. Xu Y. (2020). Brahma related gene 1 (Brg1) regulates cellular cholesterol synthesis by acting as a co-factor for SREBP2. *Front. Cell Dev. Biol.* 8:259. 10.3389/fcell.2020.00259 32500071 PMC7243037

[B11] Fan Z. Li N. Xu Z. Wu J. Fan X. Xu Y. (2019). An interaction between MKL1, BRG1, and C/EBPbeta mediates palmitate induced CRP transcription in hepatocytes. *Biochim. Biophys. Acta Gene Regul. Mech.* 1862:194412. 10.1016/j.bbagrm.2019.194412 31356989

[B12] Foster C. T. Gualdrini F. Treisman R. (2017). Mutual dependence of the MRTF-SRF and YAP-TEAD pathways in cancer-associated fibroblasts is indirect and mediated by cytoskeletal dynamics. *Genes Dev.* 31 2361–2375. 10.1101/gad.304501.117 29317486 PMC5795783

[B13] Heineke J. Molkentin J. D. (2006). Regulation of cardiac hypertrophy by intracellular signalling pathways. *Nat. Rev. Mol. Cell Biol.* 7 589–600. 10.1038/nrm1983 16936699

[B14] Hofmann F. (2018). A concise discussion of the regulatory role of cGMP kinase I in cardiac physiology and pathology. *Basic Res. Cardiol.* 113:31. 10.1007/s00395-018-0690-691 29934662

[B15] Hu X. Li T. Zhang C. Liu Y. Xu M. Wang W. (2011). GATA4 regulates ANF expression synergistically with Sp1 in a cardiac hypertrophy model. *J. Cell Mol. Med.* 15 1865–1877. 10.1111/j.1582-4934.2010.01182.x 20874724 PMC3918043

[B16] Hutchings D. C. Anderson S. G. Caldwell J. L. Trafford A. W. (2018). Phosphodiesterase-5 inhibitors and the heart: compound cardioprotection? *Heart* 104 1244–1250. 10.1136/heartjnl-2017-312865 29519873 PMC6204975

[B17] Kamo T. Akazawa H. Komuro I. (2015). Cardiac nonmyocytes in the hub of cardiac hypertrophy. *Circ. Res.* 117 89–98. 10.1161/CIRCRESAHA.117.305349 26089366

[B18] Kong M. Chen X. Lv F. Ren H. Fan Z. Qin H. (2019a). Serum response factor (SRF) promotes ROS generation and hepatic stellate cell activation by epigenetically stimulating NCF1/2 transcription. *Redox Biol.* 26:101302. 10.1016/j.redox.2019.101302 31442911 PMC6831835

[B19] Kong M. Hong W. Shao Y. Lv F. Fan Z. Li P. (2019b). Ablation of serum response factor in hepatic stellate cells attenuates liver fibrosis. *J. Mol. Med.* 97 1521–1533. 10.1007/s00109-019-01831-1838 31435710

[B20] Kuwahara K. Kinoshita H. Kuwabara Y. Nakagawa Y. Usami S. Minami T. (2010). Myocardin-related transcription factor A is a common mediator of mechanical stress- and neurohumoral stimulation-induced cardiac hypertrophic signaling leading to activation of brain natriuretic peptide gene expression. *Mol. Cell. Biol.* 30 4134–4148. 10.1128/MCB.00154-110 20606005 PMC2937559

[B21] Li Z. Chen B. Dong W. Kong M. Fan Z. Yu L. (2019a). MKL1 promotes endothelial-to-mesenchymal transition and liver fibrosis by activating TWIST1 transcription. *Cell Death Dis.* 10:899. 10.1038/s41419-019-2101-2104 PMC6881349 31776330

[B22] Li Z. Chen B. Dong W. Kong M. Shao Y. Fan Z. (2019b). The chromatin remodeler Brg1 integrates ROS production and endothelial-mesenchymal transition to promote liver fibrosis in mice. *Front. Dev. Cell Biol.* 7:245. 10.3389/fcell.2020.00245 31750301 PMC6842935

[B23] Li Z. Li P. Lu Y. Sun D. Zhang X. Xu Y. (2019c). A non-autonomous role of MKL1 in the activation of hepatic stellate cells. *Biochim. Biophys. Acta Gene Regul. Mech.* 1862 609–618. 10.1016/j.bbagrm.2019.03.001 30951901

[B24] Li Z. Lv F. Dai C. Wang Q. JIang C. Fang M. (2019d). Activation of galectin-3 (LGALS3) transcription by injurious stimuli in the liver is commonly mediated by BRG1. *Front. Cell Dev. Biol.* 7:310. 10.3389/fcell.2020.00310 31850346 PMC6901944

[B25] Li Z. Xia J. Fang M. Xu Y. (2019e). Epigenetic regulation of lung cancer cell proliferation and migration by the chromatin remodeling protein BRG1. *Oncogenesis* 8:66. 10.1038/s41389-019-0174-177 PMC6834663 31695026

[B26] Li Z. Chen B. Dong W. Xu W. Song M. Fang M. (2018). Epigenetic activation of PERP transcription by MKL1 contributes to ROS-induced apoptosis in skeletal muscle cells. *Biochim. Biophys. Acta Gene Regul. Mech.* 1861 905–915. 10.1016/j.bbagrm.2018.07.011 30056131

[B27] Li Z. Kong X. Zhang Y. Yu L. Guo J. Xu Y. (2020a). Dual roles of chromatin remodeling protein BRG1 in angiotensin II-induced endothelial-mesenchymal transition. *Cell Death Dis.* 11:549. 10.1038/s41419-020-02744-y 32683412 PMC7368857

[B28] Li Z. Zhang Y. Zhang Y. Yu L. Xiao B. Li T. (2020b). BRG1 stimulates endothelial derived alarmin MRP8 to promote macrophage infiltration in an animal model of cardiac hypertrophy. *Front. Cell Dev. Biol.* 8:569. 10.3389/fcell.2020.00569 32733885 PMC7358314

[B29] Lin H. Xiao J. Luo X. Chen G. Wang Z. (2009). Transcriptional control of pacemaker channel genes HCN2 and HCN4 by Sp1 and implications in re-expression of these genes in hypertrophied myocytes. *Cell Physiol. Biochem.* 23 317–326. 10.1159/000218178 19471099

[B30] Liu L. Mao L. Wu X. Wu T. Liu W. Yang Y. (2019). BRG1 regulates endothelial-derived IL-33 to promote ischemia-reperfusion induced renal injury and fibrosis in mice. *Biochim. Biophys. Acta Mol. Basis Dis.* 1865 2551–2561. 10.1016/j.bbadis.2019.06.015 31228616

[B31] Liu L. Wu X. Xu H. Yu L. Zhang X. Li L. (2018). Myocardin-related transcription factor A (MRTF-A) contributes to acute kidney injury by regulating macrophage ROS production. *Biochim. Biophys. Acta Mol. Basis Dis.* 1864 3109–3121. 10.1016/j.bbadis.2018.05.026 29908908

[B32] Lu Y. Lv F. Kong M. Chen X. Duan Y. Sun D. (2019). A cAbl-MRTF-a feedback loop contributes to hepatic stellate cell activation. *Front. Cell Dev. Biol.* 7:243. 10.3389/fcell.2019.00243 31681772 PMC6805704

[B33] Lukowski R. Krieg T. Rybalkin S. D. Beavo J. Hofmann F. (2014). Turning on cGMP-dependent pathways to treat cardiac dysfunctions: boom, bust, and beyond. *Trends Pharmacol. Sci.* 35 404–413. 10.1016/j.tips.2014.05.003 24948380

[B34] Mao L. Liu L. Zhang T. Wu X. Xu Y. (2020). MKL1 mediates TGF-beta-induced CTGF transcription to promote renal fibrosis. *J. Cell. Physiol.* 235 4790–4803. 10.1002/jcp.29356 31637729

[B35] McMurray J. J. Stewart S. (2000). Epidemiology, aetiology, and prognosis of heart failure. *Heart* 83 596–602. 10.1136/heart.83.5.596 10768918 PMC1760825

[B36] Minami T. Kuwahara K. Nakagawa Y. Takaoka M. Kinoshita H. Nakao K. (2012). Reciprocal expression of MRTF-A and myocardin is crucial for pathological vascular remodelling in mice. *EMBO J.* 31 4428–4440. 10.1038/emboj.2012.296 23103763 PMC3512386

[B37] Nagendran J. Archer S. L. Soliman D. Gurtu V. Moudgil R. Haromy A. (2007). Phosphodiesterase type 5 is highly expressed in the hypertrophied human right ventricle, and acute inhibition of phosphodiesterase type 5 improves contractility. *Circulation* 116 238–248. 10.1161/CIRCULATIONAHA.106.655266 17606845

[B38] Nakamura M. Sadoshima J. (2018). Mechanisms of physiological and pathological cardiac hypertrophy. *Nat. Rev. Cardiol.* 15 387–407. 10.1038/s41569-018-0007-y 29674714

[B39] Nicolas M. Noe V. Ciudad C. J. (2003). Transcriptional regulation of the human Sp1 gene promoter by the specificity protein (Sp) family members nuclear factor Y (NF-Y) and E2F. *Biochem. J.* 371(Pt 2), 265–275. 10.1042/BJ20021166 12513689 PMC1223280

[B40] Pokreisz P. Vandenwijngaert S. Bito V. Van den Bergh A. Lenaerts I. Busch C. (2009). Ventricular phosphodiesterase-5 expression is increased in patients with advanced heart failure and contributes to adverse ventricular remodeling after myocardial infarction in mice. *Circulation* 119 408–416. 10.1161/CIRCULATIONAHA.108.822072 19139381 PMC3791110

[B41] Sack M. N. Disch D. L. Rockman H. A. Kelly D. P. (1997). A role for Sp and nuclear receptor transcription factors in a cardiac hypertrophic growth program. *Proc. Natl. Acad. Sci. U.S.A.* 94 6438–6443. 10.1073/pnas.94.12.6438 9177236 PMC21068

[B42] Shao J. Weng X. Zhuo L. Yu L. Li Z. Shen K. (2019). Angiotensin II induced CSF1 transcription is mediated by a crosstalk between different epigenetic factors in vascular endothelial cells. *Biochim. Biophys. Acta Gene Regul. Mech.* 1862 1–11. 10.1016/j.bbagrm.2018.10.001 30317027

[B43] Small E. M. (2012). The actin-MRTF-SRF gene regulatory axis and myofibroblast differentiation. *J. Cardiovasc. Transl. Res.* 5 794–804. 10.1007/s12265-012-9397-9390 22898751

[B44] Sun L. Li H. Chen J. Dehennaut V. Zhao Y. Yang Y. (2013). A SUMOylation-dependent pathway regulates SIRT1 transcription and lung cancer metastasis. *J. Natl. Cancer Inst.* 105 887–898. 10.1093/jnci/djt118 23704280

[B45] Sun Y. Boyd K. Xu W. Ma J. Jackson C. W. Fu A. (2006). Acute myeloid leukemia-associated Mkl1 (Mrtf-a) is a key regulator of mammary gland function. *Mol. Cell. Biol.* 26 5809–5826. 10.1128/mcb.00024-06 16847333 PMC1592762

[B46] Takizawa T. Arai M. Tomaru K. Koitabashi N. Baker D. L. Periasamy M. (2003). Transcription factor Sp1 regulates SERCA2 gene expression in pressure-overloaded hearts: a study using in vivo direct gene transfer into living myocardium. *J. Mol. Cell Cardiol.* 35 777–783. 10.1016/s0022-2828(03)00122-126 12818568

[B47] Wang D. Z. Li S. Hockemeyer D. Sutherland L. Wang Z. Schratt G. (2002). Potentiation of serum response factor activity by a family of myocardin-related transcription factors. *Proc. Natl. Acad. Sci. U.S.A.* 99 14855–14860. 10.1073/pnas.222561499 12397177 PMC137508

[B48] Weng X. Yu L. Liang P. Chen D. Cheng X. Yang Y. (2015). Endothelial MRTF-A mediates angiotensin II induced cardiac hypertrophy. *J. Mol. Cell Cardiol.* 80 23–33. 10.1016/j.yjmcc.2014.11.009 25446178

[B49] Weng X. Zhang Y. Li Z. Yu L. Xu F. Fang M. (2019). Class II transactivator (CIITA) mediates IFN-gamma induced eNOS repression by enlisting SUV39H1. *Biochim. Biophys. Acta Gene Regul. Mech.* 1862 163–172. 10.1016/j.bbagrm.2019.01.005 30716531

[B50] Xiong Y. Bedi K. Berritt S. Attipoe B. K. Brooks T. G. Wang K. (2019). Targeting MRTF/SRF in CAP2-dependent dilated cardiomyopathy delays disease onset. *JCI Insight* 4:629. 10.1172/jci.insight.124629 30762586 PMC6483011

[B51] Yang Y. Liu L. Fang M. Bai H. Xu Y. (2019a). The chromatin remodeling protein BRM regulates the transcription of tight junction proteins: implication in breast cancer metastasis. *Biochim. Biophys. Acta Gene Regul. Mech.* 1862 547–556. 10.1016/j.bbagrm.2019.03.002 30946989

[B52] Yang Y. Liu L. Li M. Cheng X. Fang M. Zeng Q. (2019b). The chromatin remodeling protein BRG1 links ELOVL3 trans-activation to prostate cancer metastasis. *Biochim. Biophys. Acta Gene Regul. Mech.* 1862 834–845. 10.1016/j.bbagrm.2019.05.005 31154107

[B53] Yang Y. Yang G. Yu L. Lin L. Liu L. Fang M. (2020). An interplay between MRTF-A and the histone acetyltransferase TIP60 mediates hypoxia-reoxygenation induced inos transcription in macrophages. *Front. Cell Dev. Biol.* 8:484. 10.3389/fcell.2020.00484 32626711 PMC7315810

[B54] Yu L. Fang F. Dai X. Xu H. Qi X. Fang M. (2017). MKL1 defines the H3K4Me3 landscape for NF-kappaB dependent inflammatory response. *Sci. Rep.* 7:191. 10.1038/s41598-017-00301-w 28298643 PMC5428227

[B55] Yu L. Weng X. Liang P. Dai X. Wu X. Xu H. (2014). MRTF-A mediates LPS-induced pro-inflammatory transcription by interacting with the COMPASS complex. *J. Cell Sci.* 127 4645–4657. 10.1242/jcs.152314 25189621

[B56] Yu L. Yang G. Zhang X. Wang P. Weng X. Yang Y. (2018). Megakaryocytic leukemia 1 (MKL1) bridges epigenetic activation of NADPH oxidase in macrophages to cardiac ischemia-reperfusion injury. *Circulation* 138 2820–2836. 10.1161/CIRCULATIONAHA.118.035377 30018168

[B57] Zeng S. Wu X. Chen X. Xu H. Zhang T. Xu Y. (2018). Hypermethylated in cancer 1 (HIC1) mediates high glucose induced ROS accumulation in renal tubular epithelial cells by epigenetically repressing SIRT1 transcription. *Biochim. Biophys. Acta Gene Regul. Mech.* 1861 917–927. 10.1016/j.bbagrm.2018.08.002 30496037

[B58] Zhang M. Koitabashi N. Nagayama T. Rambaran R. Feng N. Takimoto E. (2008). Expression, activity, and pro-hypertrophic effects of PDE5A in cardiac myocytes. *Cell. Signal.* 20 2231–2236. 10.1016/j.cellsig.2008.08.012 18790048 PMC2601628

[B59] Zhang X. Li R. Qin X. Wang L. Xiao J. Song Y. (2018). Sp1 plays an important role in vascular calcification both in vivo and in vitro. *J. Am. Heart Assoc.* 7:e007555. 10.1161/JAHA.117.007555 29572322 PMC5907546

[B60] Zhao Q. Yang J. Chen H. Li J. Que L. Zhu G. (2019). Peli1 induction impairs cardiac microvascular endothelium through Hsp90 dissociation from IRE1alpha. *Biochim. Biophys. Acta Mol. Basis Dis.* 1865 2606–2617. 10.1016/j.bbadis.2019.06.017 31260751

[B61] Zhou N. Lee J. J. Stoll S. Ma B. Wiener R. Wang C. (2017). Inhibition of SRF/myocardin reduces aortic stiffness by targeting vascular smooth muscle cell stiffening in hypertension. *Cardiovasc. Res.* 113 171–182. 10.1093/cvr/cvw222 28003268 PMC5340142

[B62] Ziaeian B. Fonarow G. C. (2016). Epidemiology and aetiology of heart failure. *Nat. Rev. Cardiol.* 13 368–378. 10.1038/nrcardio.2016.25 26935038 PMC4868779

