# Corrigendum: An MRTF-A–Sp1–PDE5 Axis Mediates Angiotensin-II-Induced Cardiomyocyte Hypertrophy

**DOI:** 10.3389/fcell.2021.711764

**Published:** 2021-06-29

**Authors:** Teng Wu, Huidi Wang, Xiaojun Xin, Tianyi Zhang, Jie Yang, Yannan Hou, Mingming Fang, Xiang Lu, Yong Xu

**Affiliations:** ^1^Key Laboratory of Targeted Intervention of Cardiovascular Disease and Collaborative Innovation Center for Cardiovascular Translational Medicine, Department of Pathophysiology, Nanjing Medical University, Nanjing, China; ^2^Laboratory Center for Experimental Medicine, Department of Clinical Medicine, Jiangsu Health Vocational College, Nanjing, China; ^3^Department of Geriatrics, Sir Run Run Hospital, Nanjing Medical University, Nanjing, China; ^4^Institute of Biomedical Research, Liaocheng University, Liaocheng, China

**Keywords:** cardiac hypertrophy, cardiomyocyte, transcriptional regulation, angiotensin II, MRTF-A, Sp1, PDE5

Tianyi Zhang was not included as an author in the published article. The corrected Author Contributions Statement appears below.

“YX and TW conceived the project, designed the experiments, and wrote the manuscript. TW, HW, XX, TZ, JY, and YH performed experiments and collected data. MF and XL secured funding and provided supervision. All authors contributed to the article and approved the submitted version.”

The authors apologize for this error and state that this does not change the scientific conclusions of the article in any way. The original article has been updated.

